# Supplementary material for: Expression of DAZL Gene in Selected Tissues and Association of Its Polymorphisms with Testicular Size in Hu Sheep
Source: Animals (Basel). 2020 Apr 23;10(4):740. doi: 10.3390/ani10040740 (PMC7222755; doi:10.3390/ani10040740)
Supplement: Supplementary file 1 [file animals-10-00740-s001.pdf]

Expression of DAZL gene in selected tissues and association of its polymorphisms with testicular size in Hu sheep

Zehu Yuan, Jing Luo , Li Wang, Fadi Li, Wanhong Li, Xiangpeng Yue

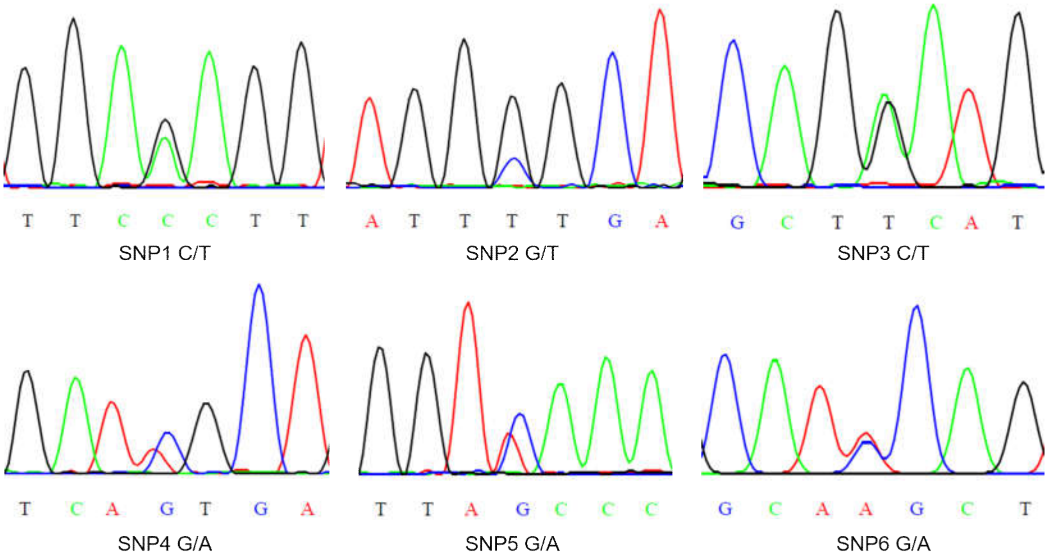


**Figure S1** the sequencing results of six single nucleotide polymorphisms (SNPs)

**Table S1** Normality test for 13 phenotypes

| Phenotype | W before transformed | P before transformed | W after transformed | P after transformed |
|---|---|---|---|---|
| LTV[1] | 0.974 | 1.376E-05 | 0.991 | 5.855E-02 |
| LTW[2] | 0.982 | 4.593E-04 | 0.995 | 4.674E-01 |
| RTV[3] | 0.978 | 7.903E-05 | 0.990 | 3.229E-02 |
| RTW[4] | 0.985 | 2.620E-03 | 0.994 | 2.988E-01 |
| LEW[5] | 0.938 | 3.227E-10 | 0.960 | 1.315E-07 |
| REW[6] | 0.971 | 5.428E-06 | 0.991 | 5.194E-02 |
| VCTW[7] | 0.802 | 2.176E-19 | 0.952 | 1.249E-08 |
| VCEW[8] | 0.838 | 1.768E-17 | 0.979 | 1.224E-04 |
| TI[9] | 0.980 | 6.000E-04 | 0.985 | 4.767E-03 |
| REW/RTW[10] | 0.863 | 4.318E-16 | 0.916 | 2.576E-12 |
| LEW/LTW[11] | 0.890 | 2.329E-14 | 0.939 | 3.986E-10 |
| TEW/TTW[12] | 0.898 | 1.064E-13 | 0.944 | 1.430E-09 |
| TTW[13] | 0.982 | 5.251E-04 | 0.996 | 4.967E-01 |

[1] left testicular volume; [2] left testicular weight; [3] right testicular volume; [4] right testicular weight; [5] left epididymis weight; [6] right epididymis weight; [7] variation coefficient between left and right testis weight; [8] variation coefficient between left and right epididymis weight; [9] testicular index; [10] right epididymis weight/ right testis weight; [11] left epididymis weight/ left testis weight; [12] total epididymis weight/ total testis weight; [13] total testicular weight.

**Table 2.** P values of Least Absolute Shrinkage and Selection Operator (LASSO) regression for 13 phenotypes.

| SNP | | | | | | Phenotype |
|---|---|---|---|---|---|---|
| **SNP1** | **SNP2** | **SNP3** | **SNP4** | **SNP5** | **SNP6** | |
| 0.693 | 0.784 | 0.860 | 0.946 | 0.550 | 0.872 | LTV1 |
| 0.548 | 0.796 | 0.838 | 0.974 | 0.568 | 0.962 | LTW2 |
| 0.588 | 0.632 | 0.672 | 1.000 | 0.412 | 1.000 | RTV3 |
| 0.710 | 0.694 | 0.716 | 1.000 | 0.439 | 0.990 | RTW4 |
| 0.877 | 0.879 | 0.367 | 0.390 | 0.917 | 0.345 | LEW5 |
| 1.000 | 0.473 | 0.036 | 0.392 | 1.000 | 0.417 | REW6 |
| 0.265 | 0.162 | 0.106 | 0.685 | 0.562 | 0.592 | VCTW7 |
| 0.288 | 1.000 | 0.761 | 0.538 | 0.585 | 0.661 | VCEW8 |
| 0.409 | 0.743 | 1.000 | 0.633 | 0.423 | 0.634 | TI9 |
| 0.651 | 0.659 | 0.826 | 0.487 | 0.573 | 0.847 | REW/RTW10 |
| 0.786 | 0.601 | 0.979 | 0.560 | 0.498 | 0.960 | LEW/LTW11 |
| 0.674 | 0.570 | 0.906 | 0.464 | 0.471 | 1.000 | TEW/TTW12 |
| 0.593 | 0.713 | 0.769 | 0.973 | 0.485 | 0.826 | TTW13 |

[1] left testicular volume; [2] left testicular weight; [3] right testicular volume; [4] right testicular weight; [5] left epididymis weight; [6] right epididymis weight; [7] variation coefficient between left and right testis weight; [8] variation coefficient between left and right epididymis weight; [9] testicular index; [10] right epididymis weight/ right testis weight; [11] left epididymis weight/ left testis weight; [12] total epididymis weight/ total testis weight; [13] total testicular weight.
